# Decentralized genomics audit logging via permissioned blockchain ledgering

**DOI:** 10.1186/s12920-020-0720-3

**Published:** 2020-07-21

**Authors:** Nicholas D. Pattengale, Corey M. Hudson

**Affiliations:** 1 Sandia National Laboratories, Albuquerque, NM USA; 2 Sandia National Laboratories, Livermore, CA USA

**Keywords:** Blockchain, Decentralized ledger, Genomics, Algorithms, Blockchain based querying

## Abstract

**Background:**

One of the tasks in the iDASH Secure Genome Analysis Competition in 2018 was to develop blockchain-based immutable logging and querying for a cross-site genomic dataset access audit trail. The specific challenge was to design a time/space efficient structure and mechanism of storing/retrieving genomic data access logs, based on MultiChain version 1.0.4 (https://www.multichain.com/).

**Methods:**

Our technique uses the MultiChain *stream* application programming interface (which affords treating MultiChain as a key value store) and employs a two-level index, which naturally supports efficient queries of the data for single clause constraints. The scheme also supports heuristic and binary search techniques for queries containing conjunctions of clause constraints, and timestamp range queries. Of note, all of our techniques have complexity independent of inserted data set size, other than the timestamp ranges, which logarithmically scale with input size.

**Results:**

We implemented our insertion and querying techniques in Python, using the MultiChain library Savoir (https://github.com/dxmarkets/savoir), and comprehensively tested our implementation across a benchmark of datasets of varying sizes. We also tested a port of our challenge submission to a newer version of MultiChain (2.0 beta), which natively supports multiple indices.

**Conclusions:**

We presented creative and efficient techniques for storing and querying log file data in MultiChain 1.0.4 and 2.0 beta. We demonstrated that it is feasible to use a permissioned blockchain ledger for genomic query log data when data volume is on the order of hundreds of megabytes and query times of dozens of minutes is acceptable. We demonstrated that evolution in the ledger platform (MultiChain 1 to 2) yielded a 30%-40% increase in insertion efficiency. All source code for this challenge has been made available under a BSD-3 license from https://github.com/sandialabs/idash2018task1/.

## Background

The development and proliferation of high-throughput genomic technologies has resulted in the fantastic exponential growth of sequenced human genomes. This growth has resulted in a doubling of the amount of genomic data every seven months [1]. It is likely that future growth in this area will primarily result from the increased amount of genomics data coming from healthcare. The Global Alliance for Genomics and Health, a standards setting body for genomics in healthcare, has estimated that as many as 60 million patients may have their genomes sequenced by 2025 [2]. Providing privacy assurances that are appropriate for protecting human health and identity information requires trade-offs between duties to share potentially lifesaving data, duties to protect privacy, and compliance with national and international electronic health information laws [3].

Work on advanced data structures and algorithms like blockchain in genomics has been put forward in order to maintain increased privacy, heighten logging and audit, and reduce the risks of centralized data storage and delivery. This work has led to developments in industry (e.g., Nebula Genomics, Gene-chain, Shivom) and distributed national healthcare [4]. One powerful characteristic of blockchain is the ability to maintain an *immutable data-access audit trail* [5]. In genomics, especially as applied to patient medical data, access audit trails for logs provide guarantees that as multiple parties (e.g., researchers, hospitals, insurance companies) access this data they create an immutable record for all queries. Since blockchains are distributed, this means that no individual party can change or manipulate logs [6]. For instance, in Institutional Review Boards, the “Final Rule” specifies the need to maintain informed consent over the like of the data, and to allow subjects to withdraw consent. Maintaining this in light of data sharing is difficult and an immutable data transaction log may help guarantee these rights are protected [7].

The National Institute of Standards and Technologies (NIST) has provided a flowchart (originally provided by Department of Homeland Security) on where blockchain technologies are expected to outperform conventional data structures. Blockchain on genomic access logs meets the standard set by NIST, in that 1) the data need to be consistently shared, 2) more than one entity contributes data, 3) data records need to be immutable, 4) sensitive identifier are not stored, 5) entities do not need to decide on control of the data, and 6) there needs to be a tamperproof log of all writes to the data [8].

In their 2018 international competition, *iDASH (integrating Data for Analysis, Anonymization, and Sharing)* posed a challenge to develop a blockchain-based immutable logging and querying for cross-site genomic dataset access audit trail. The specific challenge was to design a time/space efficient structure and mechanism for storing and retrieving genomic data access logs, based on MultiChain version 1.0.4 (https://www.multichain.com/). The details of the challenge can be found at the competition website [9].

In this paper, we describe our technique for a blockchain-based immutable logging and querying audit trail. This technique uses a straightforward two-level index. Specifically a stream (which can be thought of as a key-value store) called *logdata* maps unique keys to jsonified full log line data records, and then a stream per *logdata* column maps specific column values to *logdata* keys. This scheme supports very efficient retrieval of all records containing a particular column value, i.e. single clause queries. To generalize to the conjunction of column values (AND operations), the naïve approach would be to collect sets of IDs that match each individual column and determine the set intersection. However, MultiChain does not provide a set intersection primitive, and so the naïve approach requires fetching a large amount of data from the chain that is never returned to the user, as it is discarded during set intersection. We detail a technique for overcoming this effect via a set cardinality based heuristic, since MultiChain is able to efficiently return stream-item counts. To handle timestamp range queries, we employ a binary search filtering strategy that leverages the challenge guarantee that records are pushed to the ledger monotonically increasing by timestamp, as MultiChain otherwise does not contain a notion of sorting or ordering. Two-level indices are a standard approach to the data retrieval problem. Given that this is the first year of the iDASH log-chain track, it is therefore not surprising that standard approaches performed as well as they have. Future efforts will likely result in the growth and development of sophisticated retrieval algorithms. Furthermore, because of MultiChain’s lack of ordering, our range query was able to achieve complexity based on binary search, which is a major improvement to its out-of-box capabilities.

### Related work

The spread of blockchain technology to increasingly novel domains has led to a rapidly increasing body of related work in recent years. One particularly interesting application, which is directly relevant to the work presented in this paper is the development of secure and transparent audit logs [10]. Another related work is the development of forkable applications for blocks - which will invariable become increasingly relevant as challenges and audits to the chain become routine [11]. As the MultiChain White Paper reports, blockchain is typically not optimized for handling queries [12]. Sutton and Samavi found that audit query of a log-chain was extremely inefficient due to data structures designed for verifiability, rather than query [13]. One area that researchers are still wrestling with is, how to deal with access anonymity. This is of particular interest when access needs to be audited [14]. Our implementation focuses specifically on performance of query in audit logs, using an approach that nonetheless guarantees logs will not be overlooked or missed during query. A similar application to the one provided is the use of blockchain in the securing, standardization and simplification of electronic health records. Ledgers are currently being studied as a tool for tracking and providing guarantees that Personal Health Information logged by Internet of Things devices have trackable and auditable records [15]. Others have advocated using blockchain ledgers to guarantee secure and compliant sharing of data across clinical settings, these settings may differ in security, privacy protections and interoperability - but a ledger can potentially be used to validate and facilitate that data sharing [16].

With genomic insertion and query events a log of the creation, deletion, viewing and modification of records is essential to protect the integrity of data that have been stored - whether in blockchain or a more conventional database [17]. Electronic health records are a rich, but relatively small dataset. The unique domain where we specifically address genomic issues, using blockchain, is in the auditing of access logs. This application is essential to determining efficient access controls of sensitive genomic data. The iDASH competition provided us with the opportunity to identify and provide an efficient solution in this under-studied application of blockchain.

### Our contributions

While our implementation strategy is relatively straightforward, it was a very competitive iDASH submission performance-wise, indicating a dearth of work in data structures and algorithms promoting ease of query. Regarding novelty, 1) our multiple column constraint heuristic was unique (across iDASH submissions), non-obvious, and very well-performing in practice, and 2) as MultiChain 1.0.4 streams have no notion of sorting, our binary search method for timestamp range queries was highly creative, and asymptotically superior to all iDASH submissions of which we are aware.

## Methods

The techniques described in this paper were developed in order to submit to the blockchain-based immutable logging and querying track of the 2018 international competition *iDASH (integrating Data for Analysis, Anonymization, and Sharing)*. The specific challenge was to design a time/space efficient structure and mechanism for storing and retrieving genomic data access logs for cross-site genomic auditing, based on MultiChain version 1.0.4 (https://www.multichain.com/). The details of the challenge can be found at the competition website [9].

Submissions were evaluated according to speed, storage/memory cost, and scalability. We independently characterize our submission according to these same criteria in “Empirical evaluation” section.

### Implementation

All source code for this challenge has been made available under a BSD-3 license from https://github.com/sandialabs/idash2018task1/. Insertion and query code was written in Python, and interacts with MultiChain via the Savoir Python bindings (https://github.com/dxmarkets/savoir). Our implementation uses the MultiChain stream application programming interface (API). This API affords treating MultiChain as an append-only key-value data store (conceptually much like Redis or Amazon DynamoDB). For each log line we insert the stream items as shown in Table 1 (e.g. {1522000126703 2 28 17 3 FILE_ACCESS GTEx}). Let us assume, without loss of generality, that these insertions originate from node 2. Insertion is performed via the createrawsendfrom API, which affords inserting into multiple streams in a single API call. This data structure is sufficient to record access to a genomic record - including the time, user id, resource and activity. This specifically logs data that is critical in tracking and auditing activity on shared genomic resources.

**Table 1:** Key/values inserted for one log line

stream	key	value
logdata	UID_2_28	{timestamp:1522000126703,
		node:2,
		id:28,
		ref-id:17,
		user:3,
		activity:FILE_ACCESS,
		resource:GTEx}
timestamp	TIMESTAMP_1522b000126703	UID_2_28
node	NODE_2	UID_2_28
id	ID_28	UID_2_28
ref-id	REF-ID_17	UID_2_28
user	USER_3	UID_2_28
activity	ACTIVITY_FILE_ACCESS	UID_2_28
resource	RESOURCE_GTEx	UID_2_28
node2timestamps	1522000126703	

#### Performance and underlying algorithm

This insertion scheme has constant time and space complexity, and was chosen to afford efficiency in the various queries required by the problem statement. Specifically, the complexity of queries, depending upon mode of query are (where *n* refers to the number of log lines inserted, and *m* refers to the number of log lines returned by a query):

- Queries that contain a single constraint (i.e. column value) and no timestamp range, have complexity $\mathcal {O}(m)$
- Queries that contain multiple constraints and no timestamp range, have complexity $\mathcal {O}(|S_{i}| \geq m)$ (*S*_*i*_ detailed in “Multiple column constraints” section)
- Queries that contain timestamp ranges have complexity $\mathcal {O}(max(m,S_{i}) + log n)$

Querying is also required to support sorting by any column value. Our implementation builds a Python list of all records to return and then sorts using Python’s sorted built-in.

Our insertion scheme indexes each log line by each of its column values, and requires a stream per index. MultiChain 2, which was out of bounds for the challenge, introduced a feature allowing indexing via multiple keys. After the competition, we ported our implementation to use MultiChain 2, and compared its performance to our challenge submission.

#### Single constraint query

The insertion scheme intentionally affords a trivial method to retrieve all records containing a column with a particular value. For example, to retrieve all records with the ACTIVITY FILE_ACCESS, it would be necessary to query the activity stream (index) as follows: liststreamkeyitems activity FILE_ACCESS, which returns a collection of the identifiers, e.g. [UID_3_45, UID_2_22, UID_2_87, …, UID_2_101], which in turn are used to retrieve the records themselves (also via liststreamkeyitems). The complexity of this class of queries is $\mathcal {O}(m)$, where *m* denotes the number of records returned. When retrieving the actual records (e.g. liststreamkeyitems logdata UID_3_45), we employ a thread-pool approach via Python’s multiprocessing library, as the challenge specs indicated that Virtual Machines would have 2 cores. This does not change the complexity of the scheme, but yields noticeable performance improvements (a multiplier, roughly equal to the number of cores) in practice. As this process is trivial, we do not include pseudocode (as compared to “Multiple column constraints” and “Timestamp range constraints” sections).

#### Multiple column constraints

In order to support queries with multiple column constraints, the naïve approach would be to gather all of the subsets of UIDs per the method described in “Single constraint query” section, and then perform a set intersection. In practice, this is a poor approach due to retrieving but ultimately discarding (via the set intersection operation) large numbers of UIDs. Instead, we employ a heuristic to avoid as many unnecessary retrievals from the blockchain as possible. We observe that the result set is necessarily a subset of the smallest cardinality set over each of the column constraints. More formally, if *S*=*S*_1_∩*S*_2_∩…∩*S*_*k*_ for column constraints 1,2,…,*k*, then it follows (by the properties of set intersection) that *S*⊆*S*_*i*_ s.t. |*S*_*i*_| is smallest. We use the MultiChain API call liststreamkeys to retrieve set counts (constant time, as MultiChain caches stream size, and makes it available via constant time) instead of sets themselves (linear in the size of the returned set). Once *S*_*i*_ has been identified (by its minimum size), the actual records for *S*_*i*_ are retrieved via liststreamkeyitems. Unfortunately, this may result in records being retrieved that do not match all of the constraints (i.e. *S*⊆*S*_*i*_), and so constraints are validated, and records that do not meet all constraints are discarded. The complexity of this class of queries is *O*(|*S*_*i*_|). Pseudocode for this case is shown in Algorthm 1.


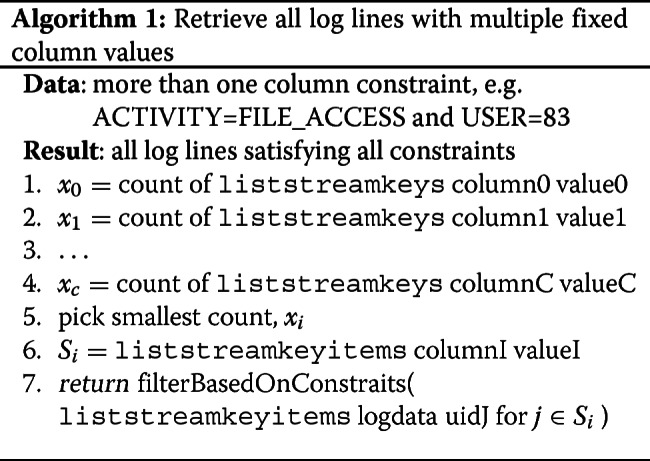


#### Timestamp range constraints

Other than the streams (indexes) we have per column, we have an additional four streams called nodeXtimestamps for *X*=1,2,3,4. The streams exist to support the requirement for timestamp range constraints in queries. The challenge rules stated that insertions would be monotonically increasing by timestamp, per node, and that records with NODE=X would only ever be inserted from node *X*. As such, we can use the MultiChain API option for liststreamkeyitems called local-ordering, which guarantees that keys can be iterated (and looked up via position) in sorted order. We wrap this with a binary search strategy, which affords finding the *UID*_*X*_*min* and *UID*_*X*_*max* in *O*(*logn*) time, where *n* is the number of inserted records. This is the only query constraint that brings into play complexity proportional to the number of inserted records. Pseudocode for this case is shown in Algorithm 2.


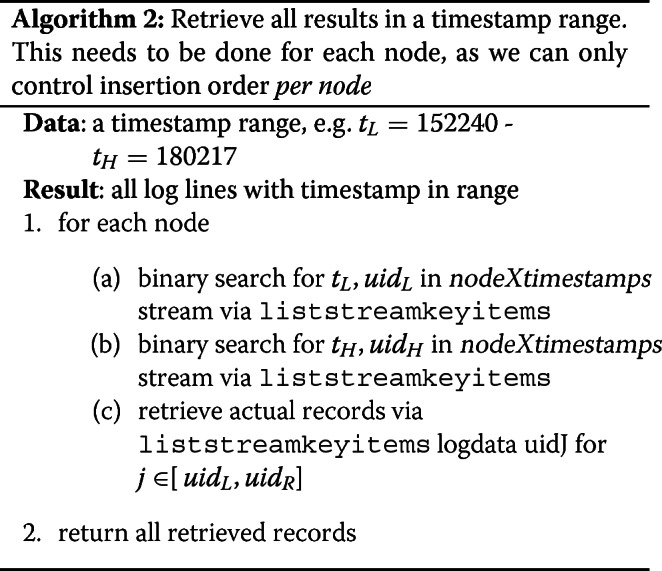


#### Software overview

**Fig. 1:**
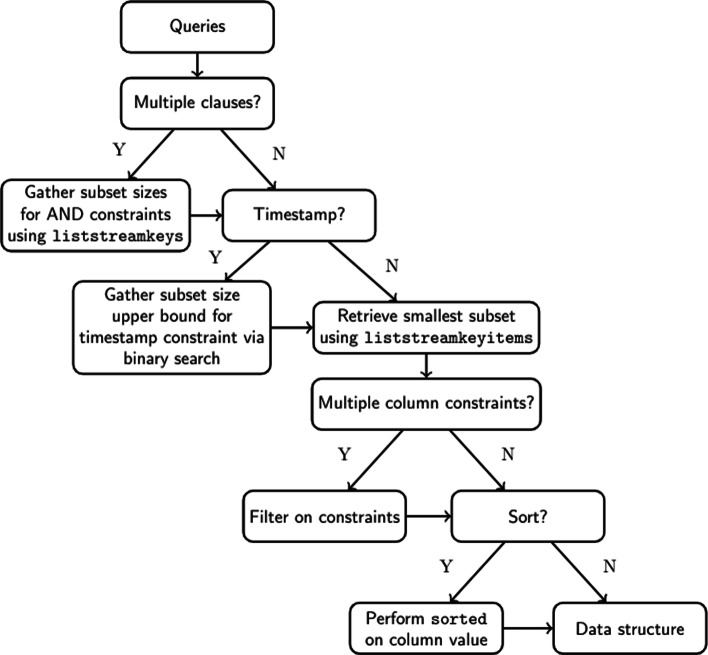
Control flow of query processing. Complex queries follow logical parsing steps indicated in this decision tree. Based on the presence of multiple clauses, timestamps, multiple column constraints and sorting, the liststreamkeys, a binary search of timestamps, filtering of constraints and the sorted functions are used to prepare queries before the data structure is delivered


Using these three approaches, depending upon the constraints expressed in any particular query, leads to this high level breakdown of our implementation (Fig. 1) and (Algorithm 3).

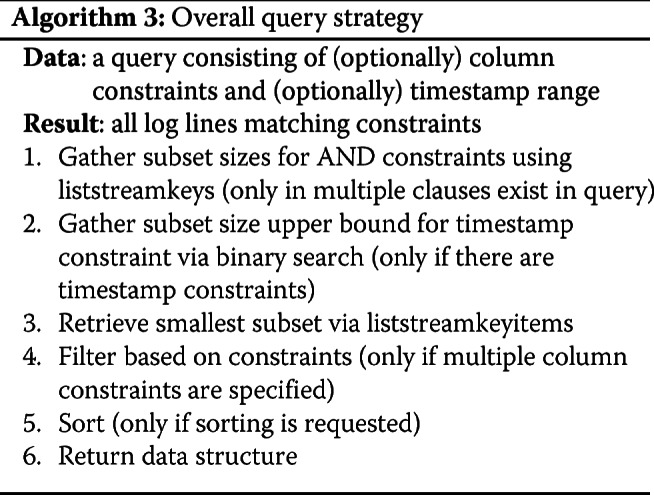


## Results

### Challenge results

The iDASH organizers evaluated our implementation by running it across three datasets. The ‘small’ dataset had 50,000 log lines per node (with a total of four nodes). The ‘medium’ and ‘large’ datasets had 100,000 and 200,000 log lines per node, respectively. Successful submissions were required to return complete and accurate responses for all test queries. Submission scores were then assigned based on a formula that took into account insertion (35% of score) and (average) query times (35% of score), as well as disk utilization (15% of score), and maximum memory consumption (15% of score).

Our iDASH submission proved highly competitive, indicating that our approach should be considered as one of the best performing implementations for this problem at the time of submission and judging. The iDASH reported performance of our implementation is as follows. Performance for each dataset was measured for insertion, average query time, disk usage, and max memory usage. For the small dataset, insertion, query, disk, memory, was 43:09, 00:00:26, 1.438GB, 22MB. For the medium dataset, 1:23:51, 00:00:47, 2.695GB, 28MB. For the large dataset, 4:07:17, 00:01:48, 5.438GB, 41MB. Given that (for example) the medium dataset contained 20MB worth of log lines, the overall on-chain storage used by our approach (28MB) is quite reasonable given that we built multiple indices for the data.

There was an inconsistency in the reported iDASH results for insertion time of the large dataset versus all of the performance that we ever observed in our lab for the insertion step. To elaborate, we intentionally rate limited insertion to 20 records per second per node (due to observed uncertainty in MultiChain transaction bottlenecks). In our testing infrastructure we were able to achieve 50 insertions per second per node without any observed difficulty, but on the contest submission cloud virtual machines we observed occasional synchronization issues with 30 records per seconds, and we opted for a conservative approach (as submissions would be disqualified if they did not synchronize). The iDASH reported time for insertion of the large dataset is a mistranscription, as the time should have been 2h46m and not 4h07m as reported, and the testers relayed that our submission was notable in that MultiChain never had to ‘catch up’ regarding node synchronization (private conversation).

### Empirical evaluation

Our testing results validate the performance reported by the conference organizers. However, to get more comprehensive understanding of the performance of our implementation, we performed further testing over a variety of dataset sizes (including throughout development). This served to both 1) empirically evaluate how our implementation scales as a function of dataset size, and 2) serve as regression tests to ensure that bugs were not introduced into the implementation as it was refined. The latter ensured that the implementation respected rules (or clarifications of the rules) that didn’t occur in the single test data set, for example the existence of repeat timestamps.

In this section we detail the performance of insertion and querying, across datasets of size 50,000, 100,000, 200,000, and 500,000 log lines for each of the four nodes per the criteria laid out in the evaluation criteria: storage, memory, total insertion time, and average query time.

As mentioned in “Multiple column constraints” section, our heuristic for supporting multiple column constraints potentially retrieves records which are ultimately discarded. We took an ad hoc approach to measure the penalty of this effect. On our 100,000 line test dataset, the predominant behavior was for 0 records to be discarded (which is a significant improvement over the naïve method), but as many as 30,000 erroneous records were retrieved for some queries, which have a very detrimental performance penalty. Further studying the tradeoffs in this false-positive/false-negative space will undoubtedly lead to more performance efficient queries.

**Fig. 2:**
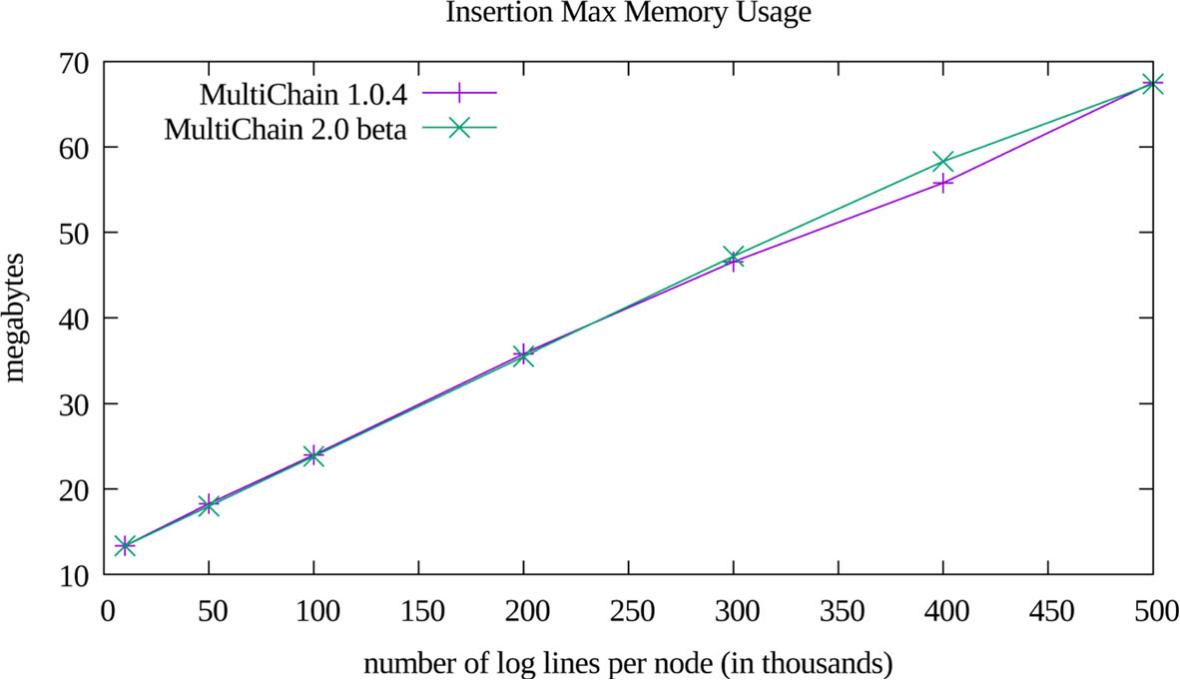
Memory Utilization. The max amount of main memory used during insertion, measured via gnu time

**Fig. 3:**
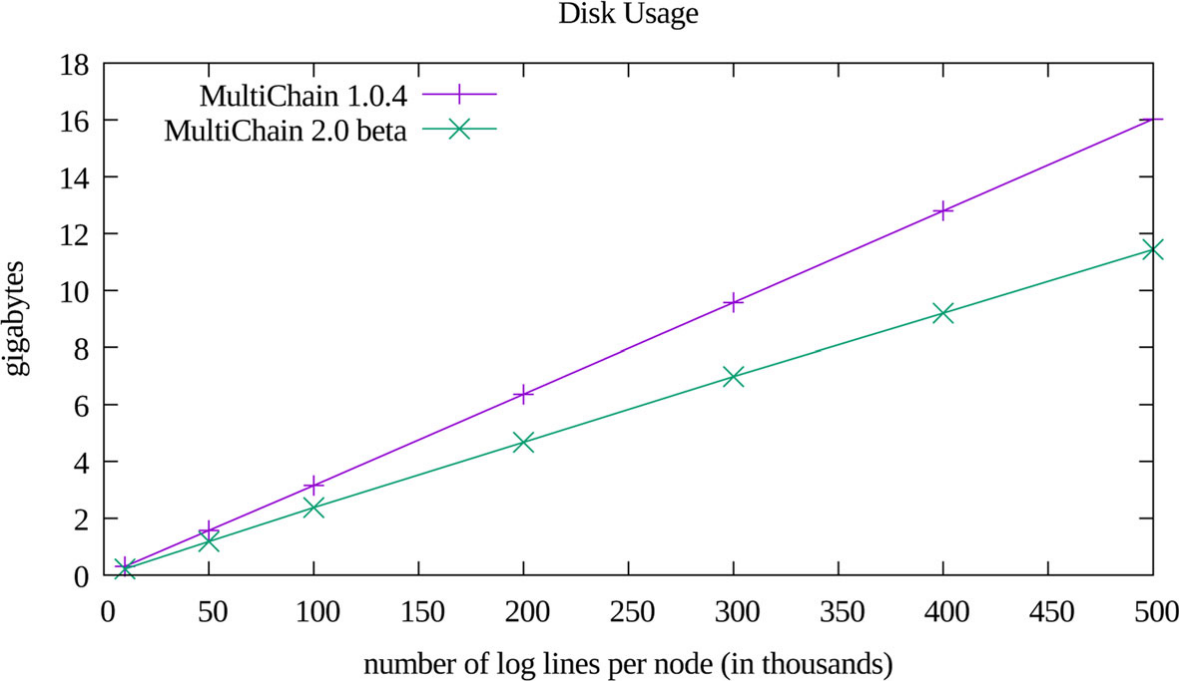
Disk Usage. The amount of storage used by MultiChain with our indexed representation. This value was calculated by measuring file system utilization before and after insertion via the Linux df command

**Fig. 4:**
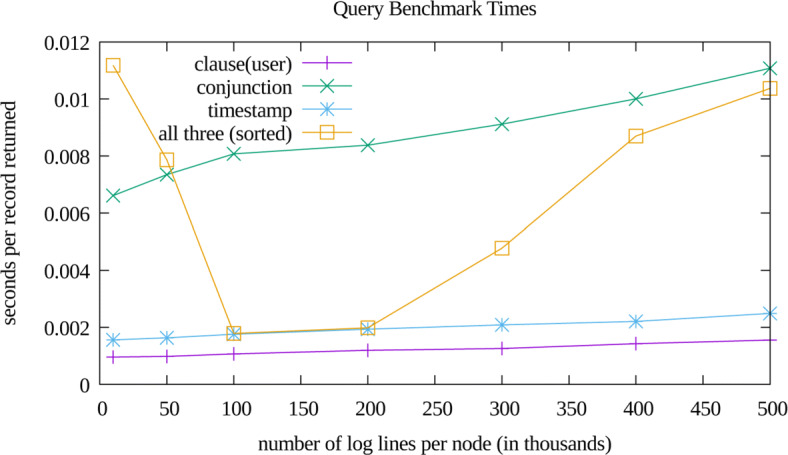
Query Scaling. A summary of query performance. Our test infrastructure, per dataset, generates a set of nine queries that cover the three query modalities (a single clause, multiple clauses, and timestamp ranges). For the nine queries, the template is fixed, but the generator chooses constraint values randomly. For each dataset, we generated five of these nine query benchmarks, and averaged the observed query times across the five samples. For this plot we have shown four of the nine query types. Examples of the four queries displayed are as follows: “QUERY user=5” clause(user), “QUERY resource=resD activity=activityE” conjunction,“QUERY timestamprange=[99171676,102561181]” timestamp, and “QUERY user=6 timestamprange=[32226847,82574461] sortby=Ref-ID” all three(sorted)

The performance of our implementation is displayed in Figs. 2,3, and 4. The memory usage and disk usage show scaling linearly proportional to log file size, which was expected. After the iDASH workshop we rapidly prototyped a port of our implementation to MultiChain version 2.0 beta, which had added native support for multiple indices (which obviated the need for our two level index). The performance of this port is only notable in the disk usage plot (Fig. 3), and did not significantly affect memory usage or query performance. The non-monotonic performance of query times as a function of dataset size (Fig. 4) likely arises due to retrieving and subsequently discarding records (per the multi-clause heuristic described in “Multiple column constraints” section). Additionally, it appears that for the tested dataset sizes, that the worst case logarithmic (as a function of inserted records) complexity of timestamp range queries is still dominated by the constant time multi-clause heuristic. The reason we did not more thoroughly explore these various confounding factors is two-fold: 1) query time is highly dependent upon the number of returned records, and 2) constructing a sufficiently wide variety of test datasets was time prohibitive for the time we had to contribute to our challenge submission. Future work will include more thoroughly examining the factors that confound straightforward empirical analysis of query efficiency.

Given that the MultiChain 2 port exhibited a 30%-40% storage savings, we could likely have *safely* increased our insertion rate to 30 records per second without risking chain islanding, resulting in a similar savings in insertion time.

### Testing infrastructure

We leveraged Sandia’s Emulytics [18] capability in building our test infrastructure. Emulytics affords precise specification of (in this case, virtualized) test networks, including granular expression of network topology, operating system type and version, and host virtual hardware and software provisioning. As such, once we specified the test network topology - a flat topology, four nodes, Ubuntu 14.04, each with two cores, and 8GB of RAM - we could effortlessly (push button) redeploy a clean network, programmatically install a clean MultiChain, programmatically install our software, run a benchmark, etc., all of which was instrumented with the automated ability to measure memory, disk, etc., usage. This proved invaluable both in development and testing, both for regression testing and benchmarking.

### Further optimizations: Z-ordering

An unsuccessful implementation strategy we attempted, applying so-called Z-ordering [19], could have been viable in slightly different challenge conditions. In Z-ordering, data with multiple keys have their keys combined (e.g. by interleaving bits), resulting in a single key space. To query for items, the key space is searched via a creative traversal strategy. In practice this approach can often outperform standard two-level indices by an order or magnitude or more. For Z-ordering to be a viable approach on a problem similar to that in iDASH 2018, there would likely have to be tighter constraints on the values of columns. In other words, if the enumerated types (activity, resource) were constrained to a small number of values (e.g. 16 or 32), and the integer types (user, id ref-id) were limited to e.g. 16 bits, then Z-ordering would likely be very efficient search procedure for this problem. We leave exploring Z-ordering in this context as future work.

## Discussion

It is worth noting that the space of permissioned blockchain ledger software is rapidly evolving, and it would not be surprising if many of the results presented here are obviated by advancements in the underlying ledger platform. This effect was somewhat observed in this challenge, where MultiChain 2.0 (which was released after the challenge was issued and had decided upon version 1.0.4) yielded an easy 30%-40% performance increase for record insertion.

## Conclusion

We described our technique for a blockchain-based immutable logging and querying audit trail. We presented creative and efficient techniques for storing and querying log file data in MultiChain 1.0.4 and 2.0 beta. We have implemented these techniques, and thereby demonstrated that it is feasible to use a permissioned blockchain ledger for genomic query log data when data volume is on the order of hundreds of megabytes and query times of dozens of minutes is acceptable. This approach allows querying parties to query immutable cross-site genomics logs (e.g., GTEx) in an efficient way with guarantees that all user activity is reported.

We have identified a number of areas for future exploration. First, we would like to look deeper into some of the observed query performance characteristics. Second, we would like to consider refinements to our multi-clause constraint heuristic, as the penalty for retrieving and subsequently discarding records is severe. Finally, we would like to explore Z-ordering as an efficient index for the situation when the same challenge problem has tighter column value constraints.

## Data Availability

All source code for this challenge has been made available under a BSD-3 license from https://github.com/sandialabs/idash2018task1/.

## References

[1] Stephens ZD, Lee SY, Faghri F, Campbell RH, Zhai C, Efron MJ, Iyer R, Schatz MC, Sinha S, Robinson GE (2015). Big data: astronomical or genomical?. PLoS Biol.

[2] Birney E, Vamathevan J, Goodhand P. Genomics in healthcare: Ga4gh looks to 2022. BioRxiv. 2017:203554.

[3] Vos S, van Delden JJ, van Diest PJ, Bredenoord AL (2017). Moral duties of genomics researchers: why personalized medicine requires a collective approach. Trends Genet.

[4] Raisaro JL, Troncoso-Pastoriza J, Misbach M, Sousa JS, Pradervand S, Missiaglia E, Michielin O, Ford B, Hubaux J-P (2018). MedCo: Enabling secure and privacy-preserving exploration of distributed clinical and genomic data. IEEE/ACM Trans Comput Biol and bioinforma.

[5] Pourmajidi W, Miranskyy A. Logchain: Blockchain-assisted log storage. 2018. Preprint at https://arxiv.org/abs/1805.08868.

[6] Ozercan HI, Ileri AM, Ayday E, Alkan C (2018). Realizing the potential of blockchain technologies in genomics. Genome Res.

[7] Choudhury O, Sarker H, Rudolph N, Foreman M, Fay N, Dhuliawala M, Sylla I, Fairoza N, Das AK. Enforcing human subject regulations using blockchain and smart contracts. Blockchain in Healthcare Today. 2018.

[8] Yaga D, Mell P, Roby N, Scarfone K. Blockchain technology overview. Natl Inst Stand Technol. 2018. https://www.nist.gov/publications/blockchain-technology-overview. Accessed 18 Nov 2019.

[9] iDASH secure genome analysis competition. 2018. http://www.humangenomeprivacy.org/2018/. Accessed 4 May 2020.

[10] Ahmad A, Saad M, Bassiouni M, Mohaisen A. Towards blockchain-driven, secure and transparent audit logs. In: Proceedings of the 15th EAI International Conference on Mobile and Ubiquitous Systems: Computing, Networking and Services. ACM: 2018. p. 443–8.

[11] Wang S, Dinh TTA, Lin Q, Xie Z, Zhang M, Cai Q, Chen G, Ooi BC, Ruan P (2018). Forkbase: An efficient storage engine for blockchain and forkable applications. Proceedings of the VLDB Endowment.

[12] Greenspan G. Multichain private blockchain-white paper. 2015. https://doi.org/http://www.multichain.com/download/MultiChain-White-Paper.pdf. Accessed 15 May 2018.

[13] Sutton A, Samavi R. Blockchain enabled privacy audit logs. In: International Semantic Web Conference. Springer: 2017. p. 645–60.

[14] Henry R, Herzberg A, Kate A (2018). Blockchain access privacy: Challenges & directions. IEEE Security & Privacy.

[15] Griggs KN, Ossipova O, Kohlios CP, Baccarini AN, Howson EA, Hayajneh T (2018). Healthcare blockchain system using smart contracts for secure automated remote patient monitoring. J Med Syst.

[16] Luo Y, Jin H, Li P. A blockchain future for secure clinical data sharing: A position paper. In: Proceedings of the ACM International Workshop on Security in Software Defined Networks, Network Function Virtualization: 27 March 2019 Richardson, TX. Edited by ACM: 2019. p. 23–7.

[17] Anderson J. Securing, standardizing, and simplifying electronic health record audit logs through permissioned blockchain technology. PhD dissertation. Dartmouth College, Department of Computer Science. 2018.

[18] Urias V, Van Leeuwen B, Stout W, Wright B. Emulytics at sandia national laboratories. MODSIM World. 2015.

[19] Slayton J. Z-order indexing for multifaceted queries in amazon dynamodb: Part 1. 2017. https://aws.amazon.com/blogs/database/z-order-indexing-for-multifaceted-queries-in-amazon-dynamodb-part-1/, Accessed 15 July 2018.

